# Safety and efficacy of repeated crosslinking assisted by transepithelial double-cycle iontophoresis in keratoconus progression after primary corneal crosslinking

**DOI:** 10.1038/s41433-020-01365-1

**Published:** 2021-01-07

**Authors:** Huping Wu, Lan Li, Shunrong Luo, Xie Fang, Xumin Shang, Zhiwen Xie, Xianwen Xiao, Huan He, Zhirong Lin, Zuguo Liu

**Affiliations:** 1 Eye Institute and Affiliated Xiamen Eye Center of Xiamen University, Fujian Xiamen, China; 2 Fujian Provincial Key Laboratory of Ophthalmology and Visual Science, Xiamen, 361102 Fujian China; 3 Fujian Key Laboratory of Ocular Surface and Corneal Disease (affiliated Xiamen Eye Center of Xiamen University), Xiamen, 361003 Fujian China

**Keywords:** Corneal diseases, Outcomes research

## Abstract

**Objectives:**

To evaluate the safety and efficacy of repeated corneal collagen crosslinking assisted by transepithelial double-cycle iontophoresis (DI-CXL) in the management of keratoconus progression after primary CXL.

**Methods:**

A retrospective analysis was conducted in the patients who underwent repeated CXL between 2016 and 2018. These patients were treated with DI-CXL if keratoconus progression was confirmed after primary CXL. Scoring of ocular pain and corneal epithelial damage, visual acuity, corneal tomography, in vivo corneal confocal microscopy (IVCM) was performed before and at 3, 6, 12, and 24 months after DI-CXL.

**Results:**

Overall, 21 eyes of 12 patients (mean age 17.3 ± 1.9 years) were included in this study. Before DI-CXL, an average increase of 4.26 D in *K*_max_ was detected in these patients with a mean follow-up interval of (23.0 ± 13.7) months. After DI-CXL, corneal epithelial damage rapidly recovered within days. Visual acuity remained unchanged with follow-up of 24 months. When compared to baseline, significant decreases were observed in *K*_max_ (at 3 months) and K2 (at 3 and 6 months) after DI-CXL. Corneal thickness of thinnest point significantly decreased at 3 months postoperatively. When compared to baseline, no significant differences were found in any of the refractive or tomographic parameters at 12 and 24 months. IVCM revealed trabecular patterned hyperdense tissues after DI-CXL in the anterior stroma at the depth of 200 μm or more. No corneal infiltration or persistent epithelial defect was recorded after DI-CXL.

**Conclusion:**

DI-CXL is safe and effective as a good alternative in stabilizing keratoconus progression after primary CXL.

## Introduction

Keratoconus is a progressive eye disease with degeneration and reduced biomechanical stability in the cornea. The loss of stability leads to protrusion and a subsequent increase in stromal thinning, thus resulting in an irregular corneal astigmatism and impaired vision. In very severe cases, lamellar or penetrating corneal transplantation is the therapeutic option to regain vision. As a progressive corneal disease, keratoconus has a considerable impact on patient’s vision and life quality. In recent years, corneal collagen crosslinking (CXL) has been recognized as a safe and effective treatment to delay or halt further progression of KC [1–3] and can reduce the need of keratoplasty [4, 5]. During CXL, riboflavin interacts with ultraviolet A light to create crosslinking of protein fibrils followed by the formation of interchain disulfide bonds, thus arresting the progression of corneal ectasia by increasing the biomechanical stability of the cornea. Various protocols [6] of CXL have been performed and extensively investigated, showing long-term efficacy of stabilization and improvement for keratoconus. CXL has become one of the standard treatments of progressive keratoconus in the world.

On the other hand, failure and progression of keratectasia after primary CXL have also been reported [7, 8]. Although the definition and criteria of progression after CXL has been reported [9], a series of studies [10–13] revealed diverse signs of deterioration after CXL, including tomographic progression, worse ocular refractive status, and even morphological alteration observed by anterior segment optical coherence tomography or in vivo confocal microscopy. Whether repeated CXL should be performed to manage keratoconus progression needs more elucidation. So far, very few studies evaluated the safety and efficacy of re-crosslinking in keratoconus with progression after CXL. Joella et.al demonstrated [13] that repeated CXL using classic epi-off protocol may be safe and effective.

However, potential complication [14–16] using standard Dresden’s protocol, such as corneal haze, sterile corneal infiltrates, recurrent erosion syndrome, etc. should be taken into consideration, as repeated epithelial removal may double the risk of postoperative complications theoretically. Hence, epi-on protocol might be a better choice for repeated crosslinking. Iontophoresis, in which an electrical gradient is used to drive negatively charged riboflavin molecules across the intact epithelium, may further enhance riboflavin penetration in transepithelial CXL. Laboratory [17–19] and clinical studies [20] of iontophoresis have been encouraging, demonstrating increased transepithelial penetration and improvement of corneal biomechanics. Nevertheless, most studies [21–23] showed inferior results of standard protocol of iontophoresis when compared to epi-off protocol. Theoretically, two continuous cycles of standard iontophoresis allowed time for riboflavin to penetrate and diffuse more posteriorly. Some study [24] showed better outcome of transepithelial CXL assisted by two continuous cycles of iontophoresis (enhanced iontophoresis) than that by standard iontophoresis. Hence, this study is aimed to investigate the safety and efficacy of transepithelial re-crosslinking assisted by double-cycle iontophoresis (DI) in patients with keratoconus progression after a primary CXL, as well as the characteristics in visual acuity (VA), corneal tomography, and morphological alteration in the corneal stroma.

## Subjects and methods

### Subjects and criteria

A retrospective study was conducted in patients who underwent the first CXL at the Affiliated Xiamen Eye Center of Xiamen University. The progression of keratoconus after CXL was identified [25] based on the presence of two or more of the following criteria: increase in *K*_max_ value (≥1D) in the tomography map difference between two consecutive corneal tomographies over at least 6 months after the first CXL, a deterioration of VA defined as a drop of one or more lines, or any changes in the refractive astigmatism as a change of 1.0 D or above.

Eyes with corneal thickness less than 380 μm at the thinnest point were excluded. Written informed consent was obtained from patients themselves (for subjects above 18 years old) or from their parents (for subjects below 18 years old). The study and surgical protocol were both approved by the hospital’s ethics committee and were performed according to the tenets of the Declaration of Helsinki.

### Surgical procedures

For re-crosslinking, transepithelial CXL assisted by double-cycle iontophoresis CXL (DI-CXL) was performed under sterile conditions in the operating room. Topical 0.1% pilocarpine eye drops were instilled 30 min before surgery. Topical 0.5% proparacaine hydrochloride eye drops were instilled twice before surgery (every 5 min). For DI-CXL, the return electrode was affixed to the skin of frontal region, while the corneal iontophoresis electrode was attached to the cornea by a vacuum adsorption device (SOOFT, Italy). The corneal electrode was filled with approximately 0.5 mL of 0.1% riboflavin solution (Ricrolin^+^, SOOFT, Italy), which was specifically designed for iontophoresis, from the open proximal side until the stainless-steel mesh was completely immersed. After that, the device was connected to a constant current generator (I-ON XL, SOOFT, Italy) set at 1 mA current. Continuous double cycles of iontophoresis were conducted without interval. The total dose of 10 mA/10 min (enhanced iontophoresis, which is different from the standard dose [26] of 5 mA/5 min) was monitored by the generator.

After completion of enhanced iontophoresis, residual riboflavin was rinsed away. Ultraviolet A irradiation [27] of 9 mW/cm^2^ with a wavelength of 365 nm was initiated using the ultraviolet lamp system (KXL system, Avedro, USA) for 10 min using a continuous mode. The UV light was then focused on the apex of the cornea through the double red crosshair alignment laser system. During irradiation, drops of balanced solution were applied to the cornea every 1 min to keep moisture and rinse away residual riboflavin. Tobramycin and dexamethasone eye ointment (Alcon, Novartis, Switzerland) was applied to the conjunctival sac postoperatively. Subsequent treatment included 0.5% loteprednol and tobramycin eye drops four times per day and tapered over 4 weeks, topical artificial tears of 0.3% hyaluronate sodium four times per day for at least 8 weeks. No soft therapeutic contact lens was applied even when the epithelium damage was observed.

### Evaluation of ocular discomfort and postoperative recovery

The Numeric Rating Scale (NRS) system [28] was applied to capture information from the patient’s perspective and the patients were asked to rate the severity of their symptom on a 0–10 scale. When applied to ocular pain, 0 reflected no pain, and 10 reflected the worst possible pain. Higher scores indicated more severe symptom of ocular pain.

The bulbar conjunctival redness ranged from 0 to 4 was provided by the instrument (Keratograph 5 M, OCULUS, Wetzlar, Germany) through comparing the photos captured with standard pictures stored in the program. Higher scores indicated greater severity of bulbar conjunctival congestion [29].

The epithelial fluorescein staining score was graded with cobalt blue light by a masked observer. Pictures were taken with a digital camera (BQ900 with IM900 digital imaging module, Haag-Streit, Switzerland). The extent of corneal epithelial damage was scored [30] according to the following scale: 0, no staining; 1, slight punctate staining (less than 30 points); 2, diffuse punctate staining (more than 30 points but less than 100 points); 3, diffuse staining more than 100 points; 4, diffuse staining with plaque covering less than one third of the cornea; 5, diffuse staining with plaque covering more than one third but less than two third of the cornea; and 6, staining with huge plaque covering more than two thirds of the cornea.

### Ocular examinations

In the preoperative and postoperative examinations, the following parameters were accessed: uncorrected distance visual acuity (UCVA), best corrected distance visual acuity (BCVA), slit-lamp microscopy examination including corneal fluorescein sodium staining, corneal tomography and pachymetry (Pentacam HR 70900, Oculus, Wetzlar, Germany), endothelial biomicroscope (SP-3000P, Topcon, Tokyo, Japan), in vivo corneal confocal microscopy (IVCM, HRT3/Rostock Cornea Module, Heidelberg Engineering GmbH, Germany). Keratometric values (*K*_max_, K1 and K2), minimum pachymetry values, etc. were derived from the tomography data. All patients were assessed at baseline and followed up for at least 24 months postoperatively.

### Statistical analysis

The data was imported to the Statistical Package for Social Sciences (SPSS Inc., Chicago, IL, version 16.0) for analysis. Repeated measures one-way ANOVA was used for statistical comparisons among timepoints. Bonferroni correction was made for multiple comparisons. The significance level was set at <0.05.

## Results

### Demographics of the subjects

A total of 625 patients (1027 eyes) with keratoconus were treated with a primary CXL procedure in our hospital from 2011 to 2017. During follow-up, 498 patients adhered to follow-up. Of these 498 patients, 12 patients (2.41%, eight male and four female) were diagnosed as having keratoconus progression after primary CXL. These patients (21 eyes in total) with keratoconus progression after primary CXL were included in this study and received a repeated CXL procedure between July 2016 and June 2018. Demographics of patients were listed in Table 1. The mean age of the patients was (17.3 ± 1.9) years. Before re-CXL, an average increase of 4.26 D in *K*_max_ was detected in these patients with a mean follow-up interval of (23.0 ± 13.7) months. After re-crosslinking, all of these patients attended the follow-up visit for at least 24 months.

**Table 1:** Demographics of the patients before transepithelial re-crosslinking.

Patient	Sex	Age, years	Eye	Time of progression after primary CXL, months	Increase in *K*_max_, D	Protocol of Primary CXL	AC
1	M	15	OD	11	4.5	Standard iontophoresis, 9 mW/cm^2^,10 min	Y
			OS	11	2.3		
2	F	19	OS	54	8.8	Epi-off, 9 mW/cm^2^,10 min	Y
3	F	18	OD	48	13.0	Standard iontophoresis, 9 mW/cm^2^,10 min	Y
			OS	48	12.1		
4	M	17	OS	9	5.2	Transepithelial KXL system, 45 mW/cm^2^, 5 min and 20 s	Y
5	M	17	OD	16	3.8	Standard iontophoresis, 9 mW/cm^2^, 10 min	Y
			OS	16	2.6		
6	M	16	OD	16	5.5	Transepithelial KXL system, 45 mW/cm^2^, 5 min and 20 s	Y
			OS	16	3.5		
7	M	17	OD	22	4.7	Standard iontophoresis, 9 mW/cm^2^, 10 min	N
			OS	22	2.1		
8	F	15	OD	12	2.5	Transepithelial KXL system, 45 mW/cm^2^, 5 min and 20 s	Y
			OS	12	2.2		
9	F	20	OS	27	3.3	Epi-off, 9 mW/cm^2^, 10 min	Y
10	M	16	OD	19	1.9	Transepithelial KXL system, 45 mW/cm^2^, 5 min and 20 s	Y
			OS	19	1.3		
11	M	21	OD	38	3.9	Epi-off, 9 mW/cm^2^,10 min	N
			OS	38	1.9		
12	M	16	OD	15	1.7	Transepithelial KXL system, 45 mW/cm^2^, 5 min and 20 s	Y
			OS	15	2.6		

*CXL* corneal collagen crosslinking, *AC* allergic conjunctivitis.

### Slit-lamp observation and ocular discomfort

**Fig. 1:**
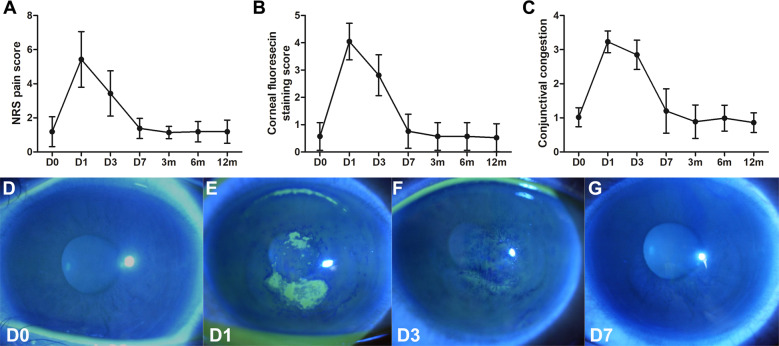
Ocular discomfort and slit lamp observation after repeated CXL. Line charts showed the NRS pain score (**a**), corneal fluorescein sodium staining score (**b**), and the conjunctival congestion score (**c**) before and after transepithelial re-crosslinking. Representative image showing the epithelial defect and recovery after re-crosslinking in one patient (**d**–**g**). On day 1 after re-crosslinking (**e**), both small plaque-like and punctual epithelial staining was seen within the central cornea (9 mm diameter).

Before surgery, no positive fluorescein staining or few punctual staining was found in the cornea. Moderate corneal epithelial fluorescein staining score and slight to moderate NRS score was observed on day 1 and 3 postoperatively, and decreased to a very low level after day 7 (Fig. 1a, b). Conjunctival congestion was apparently increased on day 1 and 3 postoperative and almost disappeared after day 7 (Fig. 1c). Representative images of corneal epithelial defect are also shown (Fig. 1d–g).

### Refractive and tomographic changes after re-crosslinking

Comparative analysis of the UCVA and BCVA as well as refractive parameters are shown in Table 2. After re-crosslinking, UCVA and BCVA slightly improved, however, no statistical difference was found when compared with the baseline. No significant difference was found in spherical dioptre, cylinder dioptre, or the spherical equivalent (*P* > 0.05), although the three parameters were slightly decreased after re-CXL.

**Table 2:** Refractive and tomographic changes after transepithelial re-crosslinking.

	Before re-crosslinking	3 months		6 months		12 months		24 months	
(*n* = 21)	Mean ± SD	Mean ± SD	*p*	Mean ± SD	*p*	Mean ± SD	*p*	Mean ± SD	*p*
UCVA	1.02 ± 0.32	1.03 ± 0.32	1.000	1.07 ± 0.38	0.524	1.04 ± 0.35	1.000	1.03 ± 0.36	1.000
BCVA	0.34 ± 0.09	0.31 ± 0.11	1.000	0.32 ± 0.13	1.000	0.36 ± 0.14	1.000	0.34 ± 0.13	1.000
Sphere, D	−3.86 ± 2.79	−3.46 ± 1.93	1.000	−3.58 ± 2.17	1.000	−3.63 ± 2.35	1.000	−3.61 ± 2.33	1.000
Cylinder, D	−3.48 ± 1.67	−2.74 ± 1.36	0.178	−2.82 ± 1.42	0.205	−3.04 ± 1.19	0.984	−2.98 ± 1.17	0.654
SE, D	−5.60 ± 2.98	−4,83 ± 2.01	0.619	−4.99 ± 2.29	0.629	−5.15 ± 2.48	1.000	−5.10 ± 2.48	1.000
K1, D	52.25 ± 4.98	52.00 ± 4.93	0.389	52.20 ± 5.18	1.000	52.39 ± 5.40	1.000	52.36 ± 5.40	1.000
K2, D	56.87 ± 4.56	56.20 ± 4.82	0.022	56.21 ± 4.90	0.036	56.55 ± 5.16	1.000	56.50 ± 5.17	1.000
*K*_max_, D	63.48 ± 6.31	62.45 ± 6.86	0.027	62.44 ± 6.80	0.179	62.59 ± 7.59	1.000	62.59 ± 7.56	1.000
Minimal thickness, μm	409.9 ± 29.7	390.4 ± 32.1	<0.001	404.3 ± 28.5	0.085	405.5 ± 26.9	0.105	405.0 ± 27.4	0.067
EC, cells/mm^2^	2668 ± 220.0	2642 ± 177.6	1.000	2641 ± 188.7	1.000	2644 ± 192.2	1.000	2644 ± 187.1	1.000
IOP, mm Hg	13.42 ± 2.03	14.00 ± 2.42	1.000	13.49 ± 1.64	1.000	13.39 ± 1.92	1.000	13.45 ± 1.88	1.000

*UCVA* uncorrected visual acuity, *BCVA* best corrected visual acuity, *SE* spherical equivalent, *EC* endothelial cell, *IOP* intraocular ocular pressure.

Corneal flattening was seen with significant decreases in *K*_max_ (at 3 months, *P* = 0.027) and K2 (at 3 and 6 months, *P* = 0.022 and *P* = 0.036, respectively) after DI-CXL. Corneal thickness of thinnest point significantly decreased at 3 months postoperatively (*P* < 0.001). When compared to baseline, no significant differences were found in any of the refractive or tomographic parameters at 12 and 24 months after DI-CXL, indicating that the keratoconus had been stabilized during 24 months of follow-up.

### Structural alteration in the corneal stroma

**Fig. 2:**
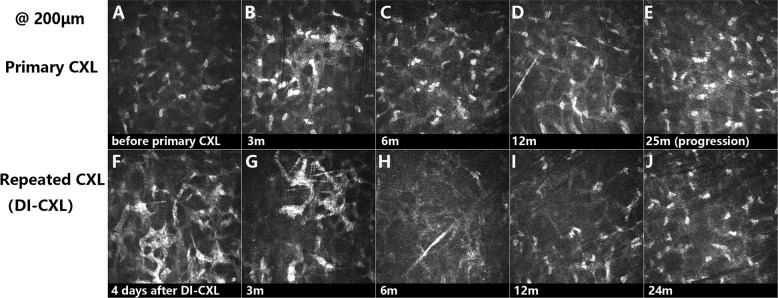
Representative images showing the structural alteration in the corneal stroma by in vivo confocal microscopy (800×). Different to the preoperative scan (**a**), lacunar oedema was visible in the first three postoperative months after primary CXL with trabecular patterned hyperdense tissue surrounding oedematous areas (**b**). After that, anterior-mid stroma was repopulated by keratocytes and surrounded by extracellular collagen tissue with slightly high density (**c**, **d**). Before re-crosslinking, hyper-reflective extracellular tissue surrounding keratocyte nuclei could hardly be seen in the anterior stroma of these patients (**e**). After re-crosslinking, lacunar oedema in the anterior stroma reappeared and could be observed during the early period postoperatively (**f**). The depth of cornea oedema could still be observed at 250 μm measured from epithelial surface. Apoptotic keratocytes and activated keratocytes with elongated membrane processes were both detectable from 3 to 6 months postoperatively (**g**, **h**). At 12 and 24 months after re-CXL, anterior-mid stroma was repopulated by keratocytes and surrounded by dense extracellular collagen tissue (**i**, **j**).

IVCM was assessed to evaluate the structural alternation in the corneal stroma. Representative images are shown in Fig. 2.

### Postoperative corneal complications

No significant endothelial cell loss or IOP elevation after DI-CXL was found. Corneal complications including corneal haze, sterile corneal infiltrates, recurrent epithelium erosion, corneal melting or perforation were not noted during the period of follow-up.

## Discussion

Long-term stability of progressive keratoconus after CXL treatment with even more than 10 years of follow-up has been shown in many studies, in which [2, 3, 31] some failures of CXL were also reported. So far, how to treat the patients with progression after a primary CXL is not extensively discussed, at least not systemically. Hafezi et al. [10] reported a repeated CXL procedure 4 years after the first CXL in one patient, and recorded a flattening effect in the cornea. Joella et al. [13] demonstrated that repeated CXL using the classic epi-off protocol, the same method in the primary CXL, might be safe and effective. Our results further support the concept that transepithelial re-crosslinking assisted by double-cycle iontophoresis is effective and safe in the treatment of keratoconus progression after primary CXL.

In experimental studies, Beshtawi et al. [32] found that human corneas receiving 2 or 3 consecutive CXL treatments within 24 h had some increase in corneal stiffness but no statistically significant difference was found when compared with the single CXL treatment group. Tabibian et al. [33] reported that the stiffness of mouse cornea was not increased after a repeated CXL performed 3 days after the first CXL. These data indicated that no more crosslinks in the anterior stroma were induced by repeating CXL within a very short period. Our IVCM results showed that crosslinks in the stroma could be further induced by a repeated procedure after several months or years. Unfortunately, the optimal surgical time of repeated CXL remains unclear. In addition to deterioration of refractive and tomographic data, IVCM image showing the decrease of crosslinks might be considered as one of the indications of repeating CXL. However, the role of IVCM in the diagnosis and treatment of keratoconus progression needs further investigation [34].

Due to the limited number of recruited subjects, it was difficult to compare the effect of different protocols (epi-on vs. epi-off, etc.) of repeated CXL. Our data showed an improvement of tomographic reading including *K*_max_ and K2 at 3 and 6 months after DI-CXL. However, at 12 and 24 months after re-crosslinking, no statistically significant difference was found when compared with the baseline. This result might indicate that the corneal remodeling at 12 and 24 months after repeated CXL was being weakened. One possible reason for the weakening of crosslinks was that CXL effect by epi-on protocol was not as good as that by epi-off protocol [35], although the penetration depth of DI-CXL could be 250 μm, which was close to that in classic Dresden’s protocol. Another reason might be that most of the included subjects were “advanced” keratoconus with progression, as the *K*_max_ values before re-CXL were greater than 58.0 D in 15 eyes (71.4%) in our study.

The limitations of epi-on CXL using iontophoresis were numerous and have been partially overcome by various modified protocols, some of which have shown better efficacy. The first limitation of iontophoresis was the fluence according to epithelial photo-attenuation or absorption of UV-A energy at 370 nm waveband. This could be compensated [36] by enhancing the fluence. The second main limitation of the iontophoresis was the oxygen consumption by the epithelium in situ limiting intraoperative oxygen diffusion. This could be compensated by using the pulsed light to UV exposure [37]. Recently, a new protocol which enhancing the fluence and application of pulsed light of UV exposure was reported and showed great outcome [38, 39]. Our results also showed double-cycle iontophoresis was effective and safe in the treatment of keratoconus progression. However, there might be limitations for a double cycle of iontophoresis that increased riboflavin concentration further limiting the oxygen diffusion and UV-A absorption and increasing the water content into the stroma thus leading to hypotony. Theoretically, high water content and stromal oedema after double iontophoresis increase the distance between collagen fibrils thus reducing crosslinks formations. However, increased corneal crosslinks and depth of crosslinking up to 25 μm were still observed by IVCM in our study.

In the past decade, the risk factors associated with primary keratoconus have been extensively discussed in plenty of studies [40–43]. However, the risk factors associated with keratoconus progression after a primary CXL remain unclear. Theoretically, the risk factors under the two conditions are comparable, such as eye rubbing [13], *K*_max_ higher than 58.0 D [11], and young age [12], etc. In our study, the history of allergic conjunctivitis was confirmed in 10 patients (83.3%) who had a habit of eye rubbing. Before re-CXL, nine eyes (42.9%) in five patients had *K*_max_ higher than 58.0  D and lower than 70.0 D, while six eyes (28.6%) in three patients represented *K*_max_ higher than 70.0 D. Eight patients (66.7%) in our study were younger than 18 years old. Therefore, patients with these risk factors need close follow-up.

## Conclusions

In conclusion, transepithelial re-crosslinking assisted by double-cycle iontophoresis could be considered as a good alternative with high safety and efficacy in stabilizing keratoconus progression after a primary CXL. However, the long-term effects of this protocol need further study.

### Summary table

#### What was known before

- Keratoconus progression after primary corneal crosslinking could be observed and repeating corneal crosslinking could be one of the solutions.
- However, repeated crosslinking procedure could increase the risk of complications.

#### What this study adds

- Transepithelial corneal crosslinking with double-cycle iontophoresis is an effective and safe protocol in stabilizing keratoconus progression after primary corneal crosslinking surgery.

## Data Availability

The datasets generated and/or analyzed during the present study are not publicly available (obtained from the affiliated Xiamen Eye Center of Xiamen University, Xiamen repository), but are available from the corresponding author upon reasonable request.
